# Regulation of neuroinflammation by matrix metalloproteinase-8 inhibitor derivatives in activated microglia and astrocytes

**DOI:** 10.18632/oncotarget.20207

**Published:** 2017-08-10

**Authors:** Eun-Jung Lee, Min-Ji Choi, Gyeongjin Lee, Bhakta Prasad Gaire, Ji Woong Choi, Hee-Sun Kim

**Affiliations:** ^1^ Department of Molecular Medicine, Tissue Injury Defense Research Center, School of Medicine, Ewha Womans University, Seoul 158-710, South Korea; ^2^ Laboratory of Neuropharmacology, College of Pharmacy and Gachon Institute of Pharmaceutical Sciences, Gachon University, Incheon 406-799, South Korea

**Keywords:** MMP-8 inhibitor, neuroinflammation, microglia, astrocytes, systemic inflammation

## Abstract

Matrix metalloproteinases (MMPs) play a pivotal role in neuroinflammation that is associated with neurodegenerative diseases. Our group recently reported that MMP-8 mediates inflammatory reactions by modulating the processing of TNF-α. To improve the efficacy of the currently available MMP-8 inhibitor (M8I), we have synthesized structurally modified M8I derivatives (comp 2, 3, 4, 5) and compared their efficacy with original compound (comp 1). Among M8I derivatives, comp 2, 3, and 5 inhibited the production of NO, ROS, and IL-6 more efficiently than the original compound in lipopolysaccharide (LPS)-stimulated microglia. When we compared the anti-inflammatory mechanisms of the most effective derivative, comp 3, with comp 1, comp 3 suppressed the mRNA expression of iNOS and cytokines more efficiently than comp 1. Although comp 1 inhibits only TNF-α processing, comp 3 additionally inhibits the expression of TNF-α. Both compounds inhibited LPS-induced activity of MAP kinases, NF-κB, and AP-1, while they increased heme oxygenase-1 expression by upregulating AMPK-Nrf2 signaling. Overall, the effect of comp 3 on anti-inflammatory signaling was much stronger than comp 1. We verified the anti-inflammatory effects of comp 1 and 3 in the LPS-injected mouse brain and primary cultured astrocytes. Comp 1 and 3 suppressed microglial activation, astrogliosis, and proinflammatory gene expression in the brain. Moreover, the compounds inhibited proinflammatory gene expression in the cultured astrocytes. Collectively, our data suggest that the MMP-8 inhibitor may be a promising therapeutic agent for neuroinflammatory disorders.

## INTRODUCTION

Microglia are resident innate immune cells in the central nervous system. They show high motility in the brain parenchyma and functionally interact with synapses. Microglia also phagocytose apoptotic cells and debris throughout the brain and release neurotrophic factors to support neuronal survival [1, 2]. Microglia are activated by various stimuli such as ageing, infection, and environmental neurotoxins and release proinflammatory mediators such as nitric oxide (NO), reactive oxygen species (ROS), cytokines, and matrix metalloproteinases (MMPs) [3, 4]. If acute inflammatory status switches to the resolution phase, it leads to tissue repair and homeostasis [5, 6]. However, prolonged and unresolved inflammation give rise to destructive chronic inflammation that results in neuronal cell death and ultimately to the onset of neurodegenerative diseases [5-7]. In addition, neuroinflammation can lead to either damage to astrocytes or astrogliosis through induction of interleukin (IL)-1 and IL-6 expression [8]. Thus, the development of agents that can inhibit chronic neuroinflammation has been suggested as an important therapeutic strategy for neurodegenerative diseases.

MMPs belong to the zinc-containing endopeptidases, which are involved in the regulation of cell-matrix composition and processing of a number of bioactive molecules [9]. Under pathological conditions, aberrant expression of MMPs contributes to cancer metastasis, arthritis, atherosclerosis, and various inflammatory and neurological disorders [10]. Our group recently reported that several MMPs are increased under neuroinflammatory conditions, and play an important role as proinflammatory mediators in the brain [11-14]. We found that expression of MMP-3, -8, and -9 is induced by lipopolysaccharide (LPS) or ɑ-synuclein treatment, and inhibition of those MMPs suppresses proinflammatory cytokines and inducible nitric oxide synthase (iNOS) in microglia [11, 12]. In addition, we demonstrated that MMP-3, -8 and -9 mediate inflammatory reactions by activating protease-activated receptor-1 in ɑ-synuclein-stimulated microglia [12]. Particularly, we reported that MMP-8, also known as neutrophil collagenase, plays a pivotal role in neuroinflammation via tumor necrosis factor alpha (TNF-α) modulation [13]. As such, MMP-8 is directly involved in TNF-α processing by converting the proform of TNF-α to the active form. Furthermore, we demonstrated that MMP-8 is a novel pathogenic factor in focal cerebral ischemia [14]. In these studies, we used a commercially available MMP-8 inhibitor (M8I) or MMP-8 shRNA to investigate the role of MMP-8. We showed that M8I specifically inhibits MMP-8 activity without affecting MMP-3 or MMP-9, which are dramatically increased in LPS-stimulated microglia [13].

When we examined the effects of the MMP-8 inhibitor (M8I) in LPS-stimulated microglia, it robustly inhibited TNF-α secretion. However, its inhibitory effect on other proinflammatory mediators such as NO, IL-6, and ROS was rather modest [13]. Therefore, in the present study, we synthesized structurally modified M8I derivatives and tested their effects in LPS-stimulated microglia in order to improve the efficacy of M8I. After selecting the best candidate compound with improved efficacy, we compared its effects and mechanism with the original M8I in LPS-stimulated microglia, astrocytes, and under neuroinflammatory conditions *in vivo*.

## RESULTS

### M8I derivatives showed anti-inflammatory effects in LPS-stimulated microglia

To find a more potent MMP-8 inhibitor compared to the original M8I (comp 1), we tested four derivative compounds (comps 2-5) with the modified functional side chains attached to the phenylsulfone ring that is known to bind to the hydrophobic MMP S1' specificity pocket [15]. Comp 1 has a methoxyl group, while comp 2, 3, and 4/5 have phenoxy, phenyl, and chlorophenyl groups attached to their phenylsulfone ring, respectively (Table 1). Comp 1, 2, 3, and 5 have a hydroxamate, while comp 4 has a carboxylate attached to the tetrahydroisoquinoline scaffold. Both the hydroxamate and carboxylate structures are known to have a zinc chelating function, which inhibits the catalytic domain of MMPs [15].

**Table 1:** Chemical structure of MMP-8 inhibitors

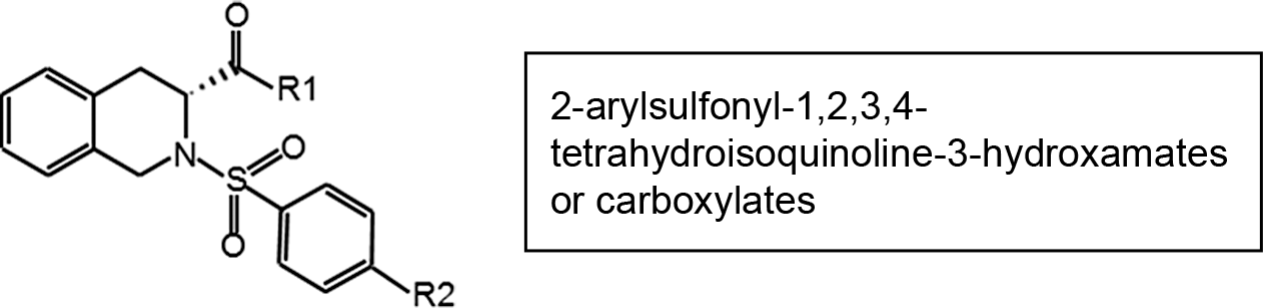
Compound	R1	R2	Chemical names
**1**	NH–OH	4-Methoxyl	(3*R*)-2-(4-methoxy) phenylsulfonyl-1,2,3,4-tetrahydroisoquinoline-3- hydroxamic acid
**2**	NH–OH	4-Phenoxy	(3*R*)-2-(4-phenoxy) phenylsulfonyl-1,2,3,4-tetrahydroisoquinoline-3- hydroxamic acid
**3**	NH–OH	4-Phenyl	(3*R*)-2-(4-phenyl) phenylsulfonyl-1,2,3,4-tetrahydroisoquinoline-3-hydroxamic acid
**4**	OH	4-(4-Chloro) phenyl	(3*R*)-2-(4-(4-chloro) phenyl) phenylsulfonyl-1,2,3,4-tetrahydroisoquinoline-3- carboxylic acid
**5**	NH–OH	4-(4-Chloro) phenyl	(3*R*)-2-(4-(4-chloro) phenyl) phenylsulfonyl-1,2,3,4-tetrahydroisoquinoline-3- hydroxamic acid

**Figure 1:**
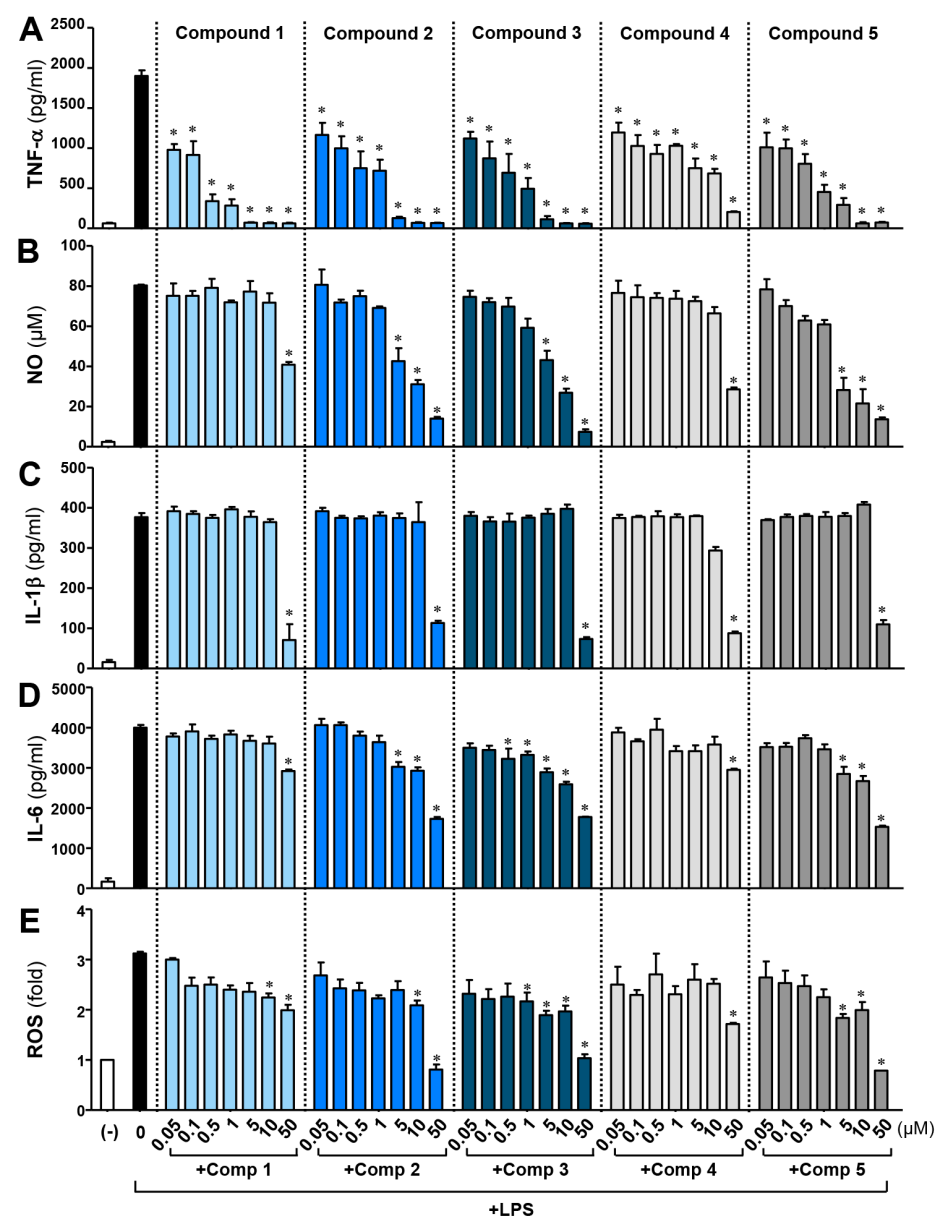
Effects of M8I derivatives (comps 1-5) on LPS-induced pro-inflammatory cytokine, NO, and ROS production in BV2 microglial cells BV2 cells were treated with lipopolysaccharide (LPS; 100 ng/ml) alone or with the indicated concentrations of comps 1-5 for 16 h. The concentrations of TNF-α **(A)**, NO **(B)**, IL-1β **(C)** and IL-6 **(D**) were measured in the supernatants. **(E)** Intracellular ROS levels were determined by measuring DCF fluorescence. Results were obtained from four independent experiments with duplicates and represent the mean ± S.E.M. *P < 0.05, significantly different from LPS-treated groups.

To compare the effects of comps 2-5 with comp 1, BV2 cells were treated with each compound for 1 h prior to LPS stimulation. As shown in Figure 1, all the compounds inhibited the production of TNF-α, NO, IL-1β, IL-6, and ROS induced by LPS. Compound 1 was the most efficient inhibitor of TNF-α secretion, followed by comp 3, comp 5, comp 2, and comp 4. However, the efficacy of NO, IL-6, and ROS inhibition was much higher for comp 2, 3, and 5 compared to comp 1. IL-1β inhibition was similar in all five compounds. The data suggest that the phenyl or phenoxy group, instead of the methoxy group, attached to the phenylsulfone ring may play a role in augmenting the inhibition of NO, IL-6, and ROS production. The compounds also have anti-inflammatory effects when administrated simultaneously or after LPS, and the anti-inflammatory effects of these compounds were confirmed in rat primary microglia (data not shown).

**Figure 2:**
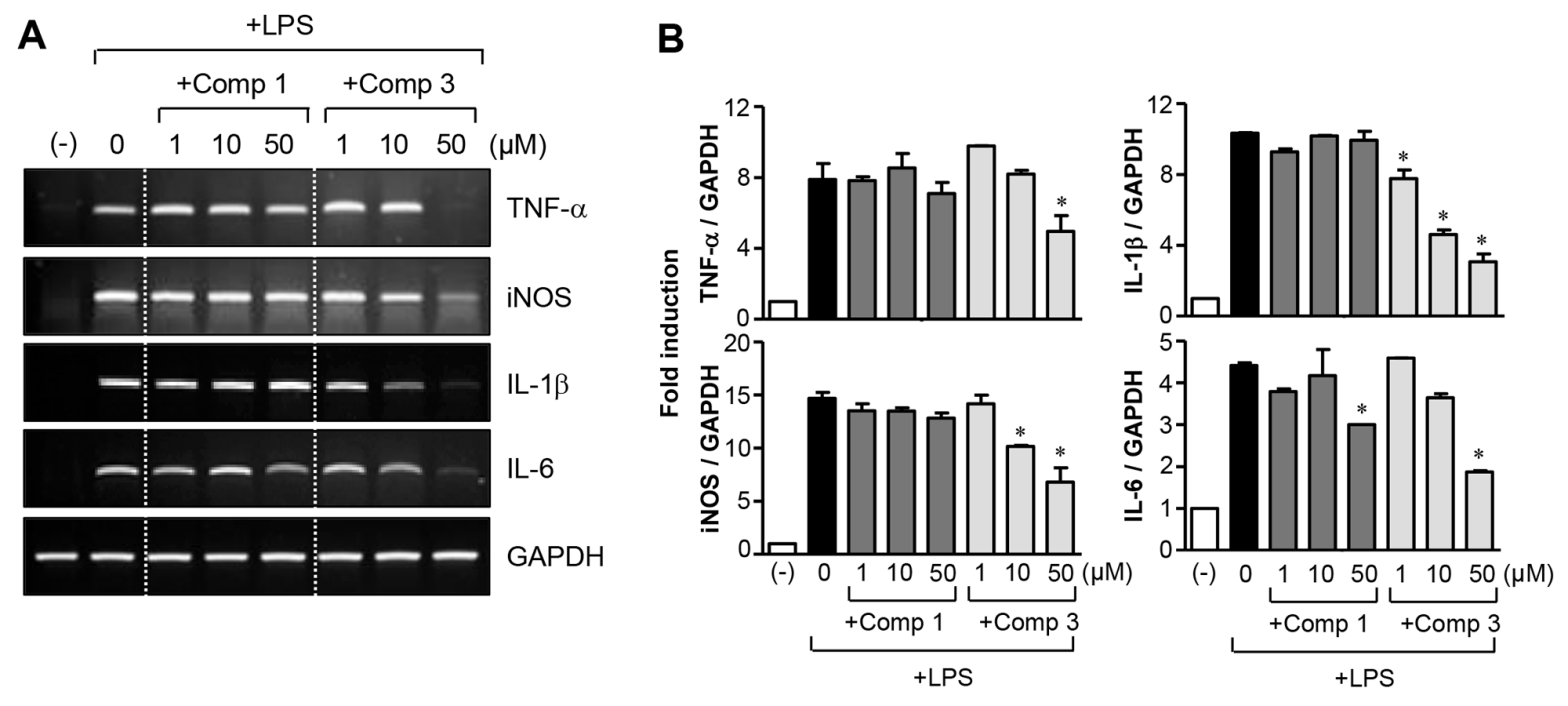
Effects of comp 1 and 3 on mRNA expression of proinflammatory molecules **(A, B)** BV2 cells were pre-treated with comp 1 or 3 for 1 h, followed by LPS (100 ng/ml) treatment for 6 h, and total RNA was isolated. The mRNA levels of iNOS and cytokines were determined by RT-PCR and normalized to GAPDH expression. Representative gels (A) and quantification of data (B) are shown (n = 5). *P < 0.05, significantly different from LPS-treated samples.

For further study, we selected comp 3 and compared its effects with comp 1 because comp 3 had the strongest anti-inflammatory effects among the M8I derivatives and showed improved efficacy of NO, IL-6, and ROS inhibition. When we examined the effects of comp 3 on the expression of inflammatory molecule mRNA, we saw that it suppressed the expression of TNF-α, iNOS, IL-1β, and IL-6 that was induced by LPS. However, comp 1 did not significantly alter their expression, except for IL-6 (Figure 2). The data suggest that comp 3 modulates the expression of iNOS and cytokines at an mRNA level.

### Comp 3 inhibited both the secretion and expression of TNF-α in LPS-stimulated microglial cells

**Figure 3:**
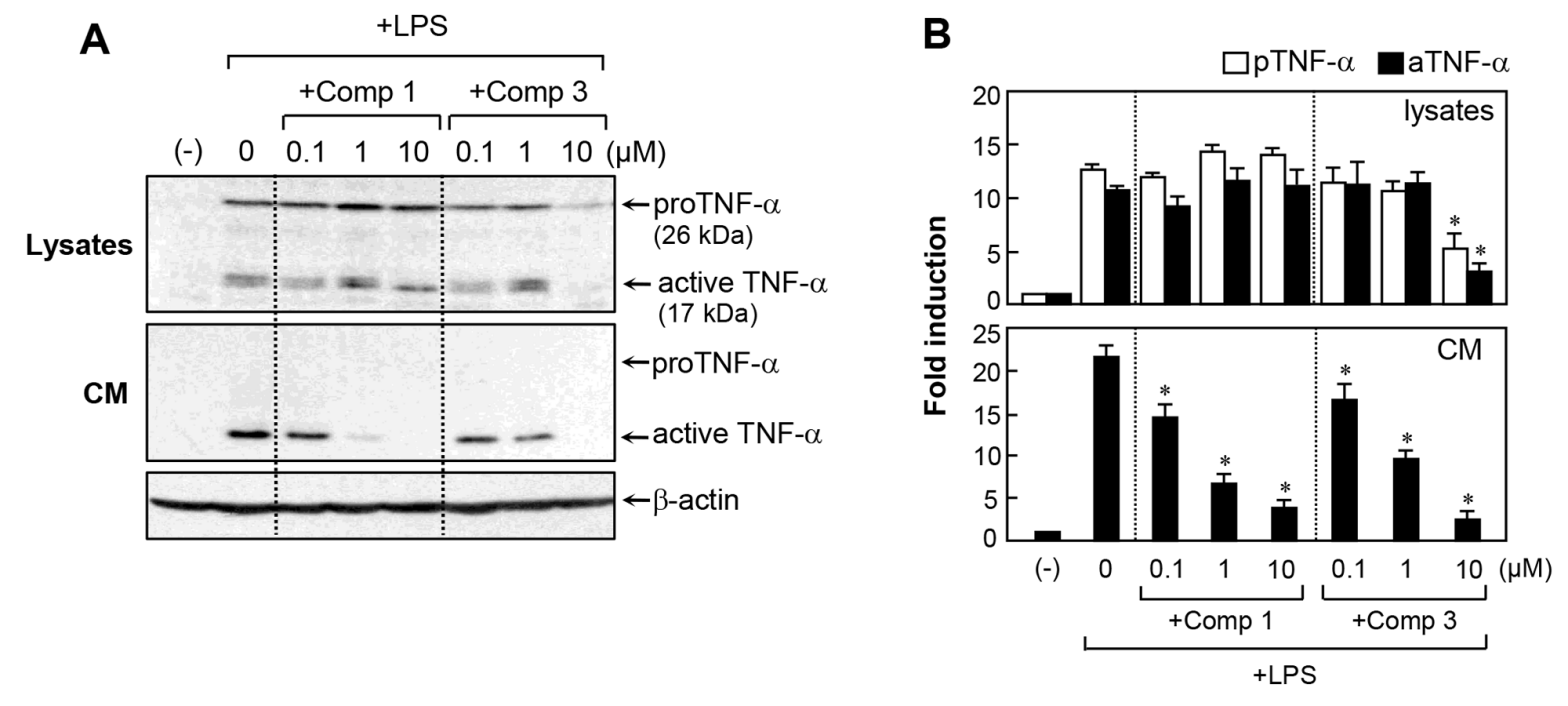
Effects of comp 1 and 3 on TNF-α expression and secretion in LPS-treated BV2 cells **(A)** Effects of comp 1 and 3 on protein expression and secretion of TNF-α were determined by western blot analysis in LPS-stimulated BV2 cell lysates and conditioned media (CM). BV2 cells were pre-treated with comp 1 or 3 for 1 h, followed by LPS (100 ng/ml) for 6 h. Representative blots showing the proform (26 kDa) and active form (17 kDa) of TNF-α are shown. **(B)** Fold change of TNF-α relative to control cells after normalization to β-actin. Results were obtained from three independent experiments and represent the mean ± S.E.M. *P < 0.05, significantly different from LPS-treated cells.

We previously reported that M8I prominently inhibits TNF-α processing in LPS-treated microglia [13]. In the present study, we compared the effects of comp 1 and comp 3 on TNF-α secretion and expression. Western blot analysis showed that comp 1 inhibited the secretion of TNF-α into culture medium without affecting protein expression (Figure 3). In contrast, comp 3 suppressed the expression of TNF-α as well as its secretion. The data suggest that comp 3 modulates TNF-α in a somewhat different manner from comp 1, probably due to the differences in their functional side chains.

### Comp 1 and 3 suppressed LPS-induced NF-κB/AP-1 activity and phosphorylation of MAPKs

**Figure 4:**
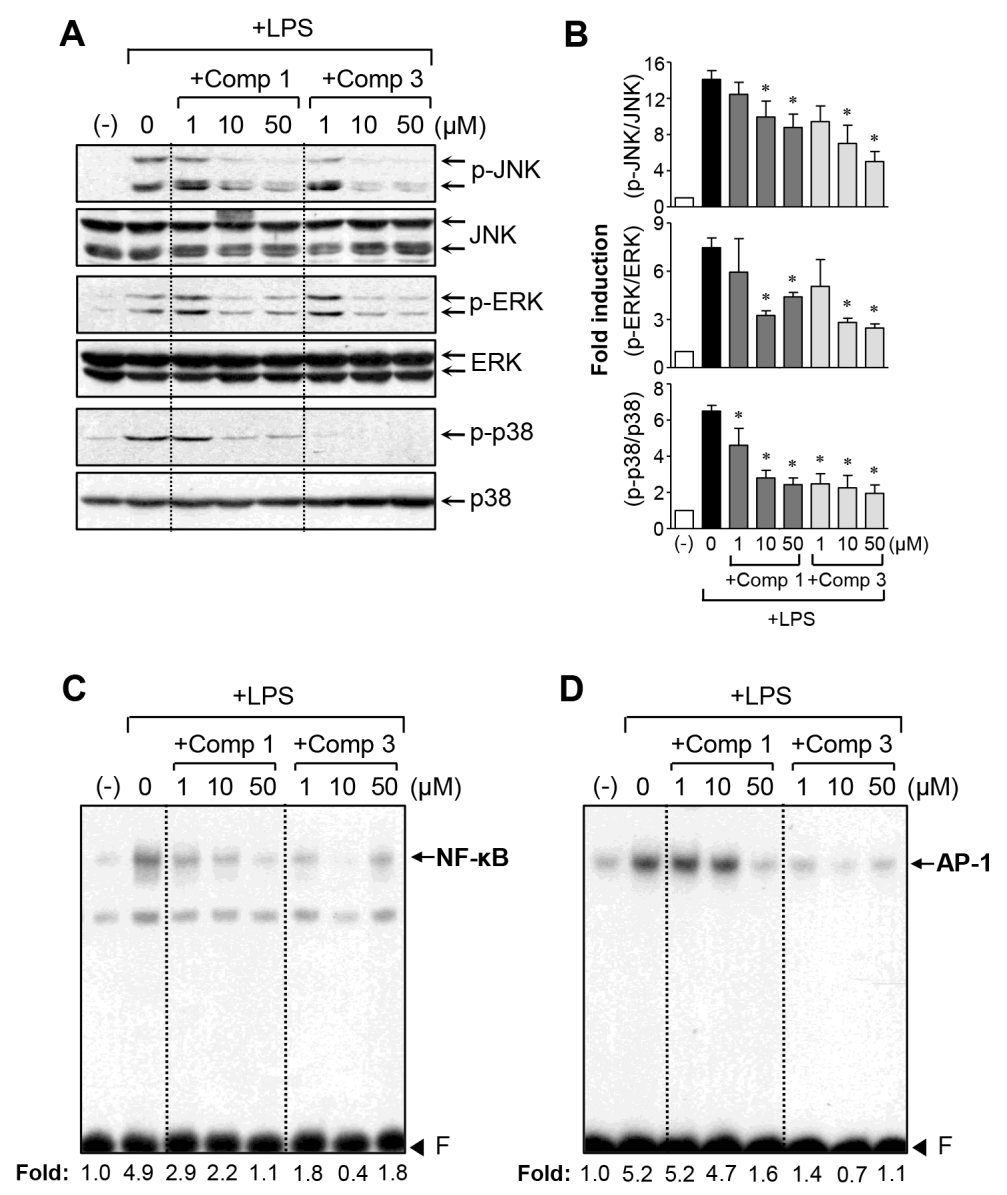
Effects of comp 1 and 3 on the phosphorylation of MAPKs, as well as NF-κB and AP-1 activity **(A)** Western blot analysis for MAPK activities. Cell extracts were prepared from BV2 cells pretreated with comp 1 or 3 for 1 h, followed by LPS (100 ng/ml) treatment for 30 min, and then subjected to western blot analysis using antibodies against phospho- or total forms of JNK, ERK, or p38 MAPK. The blots are representative of three independent experiments. **(B)** Quantification of western blot data (n=3). Levels of the phosphorylated forms of MAPKs were normalized with respect to the level of each total form and expressed as relative fold changes versus the untreated control group. *P < 0.05, significantly different from LPS-treated groups. **(C, D)** Electrophoretic mobility shift assay (EMSA) for NF-κB (C) and AP-1 (D). BV2 cells were pretreated with comp 1 or 3 for 1 h followed by LPS treatment for 3 h. The nuclear extracts were prepared from the cells and incubated with NF-κB or AP-1 probes. The arrow indicates the DNA-protein complex of NF-κB or AP-1. ‘F’ indicates a free probe.

Given that the mitogen-activated protein kinases (MAPKs) regulate the inflammatory response in microglial cells, we examined the effects of comp 1 and 3 on MAPK activity. Western blot analysis revealed that comp 1 and 3 markedly inhibited the phosphorylation of MAPKs in LPS-stimulated BV2 cells (Figure 4A, 4B). Notably, comp 3 inhibited MAPK phosphorylation more potently than comp 1. In addition, comp 1 and 3 suppressed the DNA binding activities of NF-κB and AP-1, which are essential transcription factors for pro-inflammatory gene expression [16] (Figure 4C, 4D). Interestingly, comp 3, compared to comp 1, more dramatically inhibited AP-1 DNA binding activity, which may contribute to its strong inhibitory effect on the expression of iNOS and cytokines.

### Comp 1 and 3 upregulated anti-inflammatory/antioxidant AMPK-Nrf2 signaling

**Figure 5:**
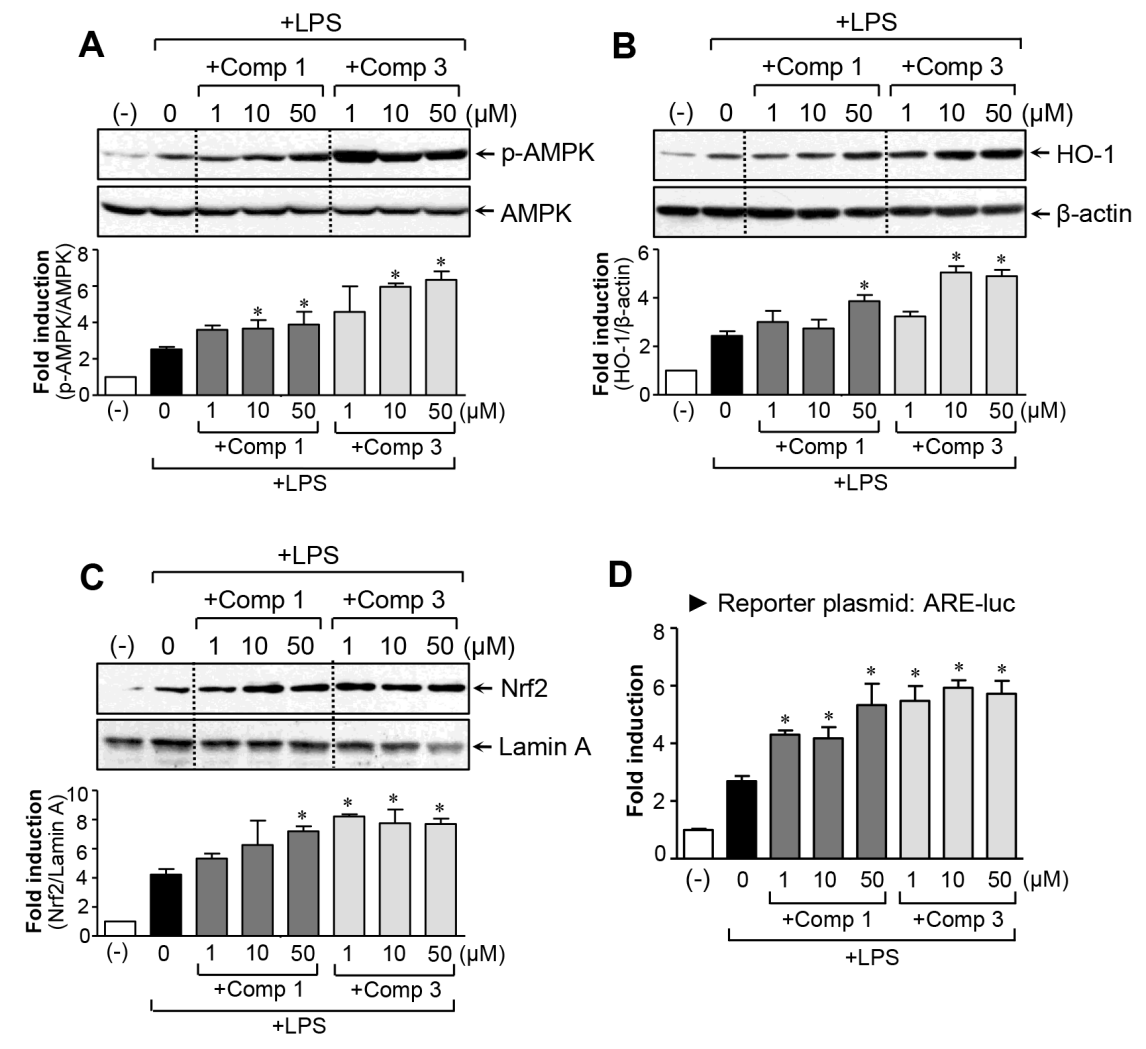
Effects of comp 1 and 3 on AMPK and Nrf2/ HO-1 signaling pathways **(A)** Cell extracts were prepared from BV2 cells treated with LPS (100 ng/ml) alone or with the indicated concentrations of comp 1 or 3 for 1 h and then subjected to western blot analysis using antibodies against the phospho- or total form of AMPK. Quantification data are shown in the bottom panel. **(B)** The effects of comp 1 and 3 on HO-1 protein expression. BV2 cells were pretreated with comp 1 or 3 for 1 h prior to incubation with LPS for 6 h. **(C)** Effects of comp 1 and 3 on nuclear translocation of Nrf2. BV2 cells were pretreated with comp 1 or 3 for 1 h prior to incubation with LPS for 3 h and then nuclear fractions were analyzed using western blot analysis. **(D)** Effects of comp 1 and 3 on ARE-luc reporter gene activity. Cells transfected with the reporter plasmid (ARE-luc) were pretreated with comp 1 or 3 for 1 h prior to incubation with LPS for 6 h, and a luciferase assay was performed. Results were obtained from four independent experiments and represent the mean ± S.E.M. *P < 0.05, significantly different from LPS-treated groups.

The AMP-activated protein kinase (AMPK) and nuclear factor-E2-related factor-2 (Nrf2) pathways are involved in the regulation of inflammation and redox signaling [17-19]. Moreover, several papers have reported that AMPK contributes to the suppression of proinflammatory reactions via Nrf2 activation [20-22]. In the present study, we found that comp 3 significantly enhanced LPS-induced AMPK phosphorylation, whereas the effect of comp 1 was rather modest (Figure 5A). Next, we examined the effects of comp 1 and 3 on Nrf2-mediated heme oxygenase-1 (HO-1) expression. Both compounds significantly increased HO-1 expression (Figure 5B). Moreover, they increased nuclear translocation of Nrf2 and antioxidant response element (ARE)-luc reporter gene activity (Figure 5C, 5D). The extent of upregulation of Nrf2/HO-1 signaling by comp 3 was greater than comp 1, as with AMPK activation. The results collectively suggest that AMPK and Nrf2/HO-1 signals may at least partially contribute to the differential anti-inflammatory and antioxidant effects of comp 1 and 3.

### Comp 1 and 3 inhibited microglial activation in the brain of mice with LPS-induced systemic inflammation

**Figure 6:**
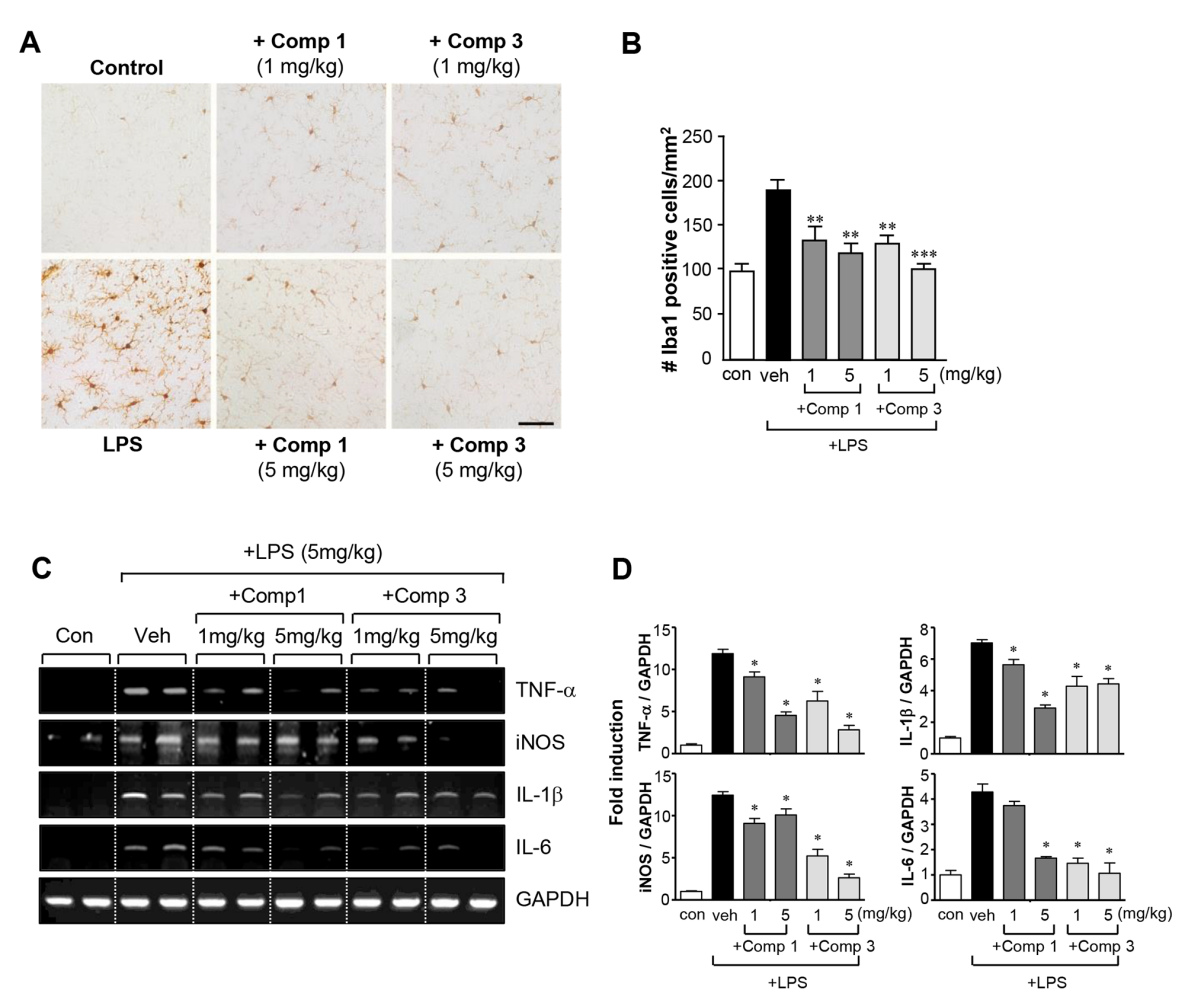
Comp 1 and 3 suppress microglial activation and the expression of pro-inflammatory molecules in LPS-induced sytemic inflammation **(A, B)** Immunohistochemical staining for Iba1 and quantification of the number of Iba1-positive microglia 3 h after systemic LPS treatment (5 mg/kg, i.p.). Microglial activation in the cortex of LPS-injected mice was reduced by comp 1 or 3 (1 or 5 mg/kg) treatment. Representative images (A) and quantification of data (B) are shown (n = 4). Scale bars, 50 μm. **(C, D)** Effects of comp 1 or 3 (1 or 5 mg/kg) on the mRNA expressions of proinflammatory cytokines (TNF-α, IL-1β, IL-6) and iNOS in the cortex of LPS-injected mice. Representative gels (C), and quantification data (D) are shown (n = 4). **P < 0.01 and ***P < 0.001, significantly different from LPS-treated mice.

To verify the anti-inflammatory effects of comp 1 and 3 under *in vivo* neuroinflammatory conditions, mice were administrated with the compound before LPS injection. Because microglia are the main players in the neuroinflammatory response, the level of microglial activation was determined using Iba1 staining. As shown in Figure 6A and 6B, administration of LPS resulted in a robust increase in the number of Iba1-positive, activated microglia, which was markedly reduced by comp 1 or 3 treatment. RT-PCR analysis showed that comp 1 and 3 inhibited the expression of iNOS and proinflammatory cytokines in the LPS-injected mouse brain (Figure 6C, 6D).

### Comp 1 and 3 suppressed astrogliosis and inflammatory responses in LPS-stimulated primary astrocytes

**Figure 7:**
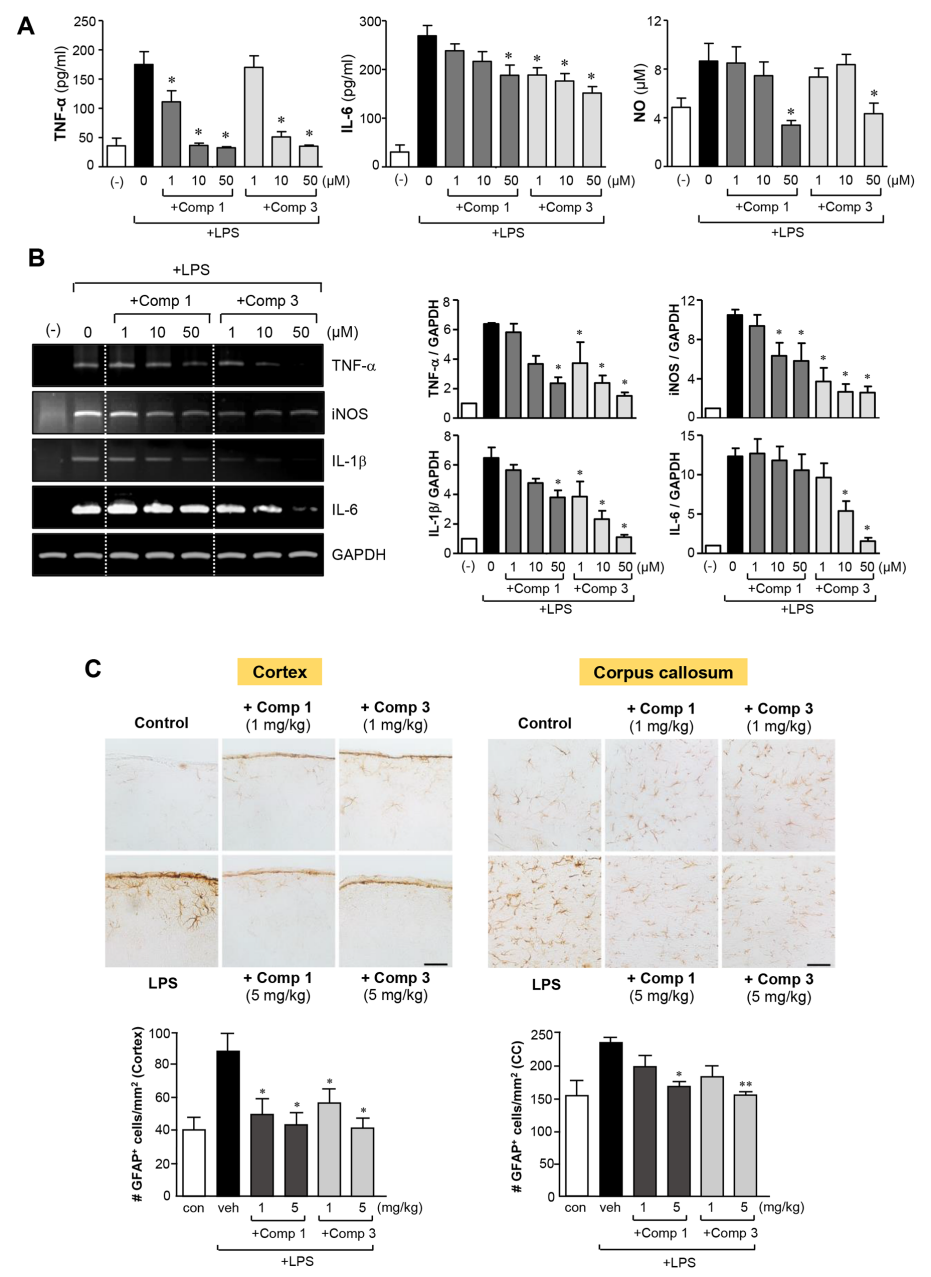
Anti-inflammatory effects of comp 1 and 3 in LPS-stimulated primary astrocytes, and inhibition of astrogliosis in septic mouse brains **(A)** Rat primary astrocytes were treated with LPS (100 ng/ml) alone or with comp 1 or 3 for 16 h. The concentrations of TNF-α, IL-6 and NO were measured in the supernatants. **(B)** To determine the effects of comp 1 or 3 on mRNA expression of iNOS and cytokines, primary astrocytes were pre-treated with comp 1 or 3 for 1 h, followed by LPS (100 ng/ml) for 6 h, and total RNA was isolated. The mRNA levels of iNOS and cytokines were determined by RT-PCR and normalized to GAPDH expression. Quantification of data are shown in the right panel (n = 3). **(C)** Effects of comp 1 or 3 on astrogliosis in the cortex and corpus callosum of LPS-injected mice. Immunohistochemical staining for GFAP and quantification of the number of GFAP-positive cells 3 h after systemic LPS treatment (5 mg/kg). Representative images (upper panels) and quantification of data (bottom panels). n = 4. Scale bars, 50 μm. Results are represented as the mean ± S.E.M. *P < 0.05 and **P < 0.01, significantly different from LPS-treated groups.

There is accumulating evidence that astrogliosis is a characteristic feature of diverse neurodegenerative pathologies and that reactive astrocytes contribute to neuroinflammation-related disease progression [23, 24]. In this study, we examined the effects of comp 1 and 3 on LPS-induced inflammatory responses in astrocytes. As shown in Figure 7A, comp 1 and 3 significantly inhibited the production of TNF-α, IL-6, and NO in rat primary astrocytes. Subsequent RT-PCR analysis showed that comp 1 and 3 suppress the expression of TNF-α, iNOS, IL-1β, and IL-6 at the mRNA level (Figure 7B). As in microglial cells, the efficacy of comp 3 was higher than comp 1.

Next, we evaluated the effects of comp 1 and 3 on astrogliosis triggered by LPS in the brain of mice with systemic inflammation. Immunohistochemistry with GFAP staining showed astrogliosis in the cortex and corpus callosum of LPS-injected mice, as indicated by the increased number of GFAP-positive cells. Moreover, cell bodies of GFAP-positive cells were larger and more densely stained compared to the control. However, treatment with comp 1 or 3 reduced the number of GFAP-positive reactive astrocytes, along with causing a reduction in size and density of cell bodies (Figure 7C). Collectively, our data suggest that comp 1 and 3 exert anti-neuroinflammatory effects by modulating the activation of astrocytes as well as microglia.

## DISCUSSION

MMPs play an important role in cancer, arthritis, atherosclerosis, and various inflammatory and neurological disorders [9, 10, 25]. Therefore, many pharmaceutical companies have searched for compounds that can inhibit their action. Most synthetic MMP inhibitors are based on a hydroxamate structure, which interferes with the action of the zinc catalytic domain in the MMP molecule [26, 27]. Additional compounds have been developed using alternative zinc-binding groups, such as carboxylic acids, thiols, and phosphorous-based groups [27, 28]. Our group recently reported that MMP-8 plays a pivotal role in neuroinflammation [13]. MMP-8 was upregulated in LPS-stimulated microglia and in the brains of mice with sepsis, and treatment with an MMP-8 inhibitor (M8I) dramatically suppressed the secretion of proinflammatory molecules, particularly TNF-α. However, the effects of M8I on other cytokines and iNOS were rather minor compared with TNF-α.

At present, to improve the efficacy of M8I, we have synthesized several M8I derivative compounds by modifying the functional group attached to phenylsulfone ring (comps 2-5), or by changing the hydroxamate to a carboxylate structure (comp 4). The hydroxamate and carboxylate have a zinc chelating function, while the arylsulfone group binds to the hydrophobic MMP S1' specificity pocket [15]. Our previous study demonstrated that M8I (comp 1) specifically inhibits MMP-8 activity without affecting MMP-3 or MMP-9, which are dramatically increased in LPS-stimulated microglia [13]. In the present study, we found that M8I derivatives (comps 2-5) also specifically inhibit MMP-8 activity in LPS-stimulated BV2 microglia (data not shown). We found that all the compounds prominently inhibited TNF-α production, ordered as follows by efficacy: comp 1 > comp 3 > comp 5 > comp 2 > comp 4. However, comp 2, 3, and 5 showed greater efficacy of NO, IL-6, and ROS inhibition compared with comp 1 (Figure 1). From these data, we conclude the following: i) comp 1 is most efficient in TNF-α inhibition, suggesting that the methoxyl group attached to the phenylsulfone ring and the hydroxamate structure are sufficient for TNF-α inhibition; ii) substitution of the methoxyl group with a phenoxy (comp 2), phenyl (comp 3), or chlorophenyl (comp 5) group improved the efficacy of NO, IL-6, and ROS inhibition, suggesting that the phenyl group may be better than the methoxyl group for NO, IL-6, and ROS inhibition; and iii) comp 4, harboring a carboxylate with a substituted chlorophenyl group, did not show improved efficacy in NO, IL-6, and ROS inhibition, and TNF-α inhibition was also lowest among the five compounds. The results suggest that the hydroxamate structure is more efficient than carboxylate for TNF-α inhibition, and the hydroxamate is also necessary for boosting the effects of the phenyl group on NO, IL-6, and ROS inhibition.

In subsequent studies, we selected comp 3, with the best anti-inflammatory effect among the derivatives, and compared its function and mechanism with comp 1. We observed that comp 3 regulated the expression of TNF-α, iNOS, IL-1β, and IL-6 at the mRNA level, whereas comp 1 inhibited only IL-6 mRNA expression (Figure 2). As we previously reported, comp 1 blocked TNF-α secretion without affecting its expression. In contrast, comp 3 inhibited TNF-α expression as well as its secretion (Figure 3). Further mechanistic studies showed that both comp 1 and 3 inhibited the phosphorylation of three types of MAP kinases, as well as the DNA binding activities of NF-κB and AP-1 (Figure 4). Interestingly, we found that the inhibitory effect of comp 3 on AP-1 binding was much stronger than comp 1, which may be one of the factors contributing to efficient inhibition of iNOS and cytokine expression by comp 3.

Despite the anti-inflammatory effects of M8I in activated microglia, antioxidant effects and impact on redox signaling by M8I have not yet been reported. In the present study, we found that comp 1 and 3 inhibited ROS production and upregulated antioxidant HO-1 expression (Figure 5). Consistent with the stronger antioxidant effects of comp 3 compared to comp 1, the fold induction of HO-1 by comp 3 was larger than comp 1. Previous studies by our and other groups reported that AMPK signaling plays an important role in mediating anti-inflammatory and antioxidant mechanisms in microglia [17, 29]. In particular, AMPK activation is involved in ROS inhibition and Nrf2/HO-1 upregulation, contributing to its anti-inflammatory and antioxidant effects in LPS-stimulated microglia [29]. In accordance with this, the present study found that comp 1 and 3 increased AMPK activation (Figure 5). The fold induction by comp 3 was much larger than comp 1. Collectively, the data suggest that AMPK and its downstream Nrf2/HO-1 signaling axis are at least partly involved in the anti-inflammatory/antioxidant mechanism of MMP-8 inhibitors.

In the present study, we found that M8I inhibits the inflammatory response in activated astrocytes as well as in microglia. Although the involvement of astrocytes in neuroinflammatory diseases has been less extensively studied than that of microglial cells, astrocytes also participate in CNS immune responses and the progression of neural pathologies, which is considered critical in the modulation of neuroinflammation [30]. Recent studies demonstrated that IL-1 and IL-6 are key players in neuroinflammation-induced astrogliosis, and astrogliosis is a characteristic feature of various neurodegenerative diseases [30, 31]. Therefore, the anti-inflammatory effects of M8I in LPS-stimulated astrocytes and the M8I-mediated inhibition of astrogliosis may contribute to the strong anti-inflammatory action of M8I in the brain of mice with sepsis.

Through this study, we have developed multiple MMP-8 inhibitor derivatives and demonstrated their anti-inflammatory and antioxidant effects in microglia, astrocytes, and systemically inflamed mouse brains. Moreover, the structure-function relationships of the M8I derivatives have been explored. We found that chemical modification of the methoxyl group attached to the phenlysulfone ring to a phenyl group improved the efficacy of the original M8I. Therefore, the strong anti-inflammatory and antioxidant effects of M8I derivatives, such as comp 3, may provide improved therapies for various neuroinflammatory disorders.

## MATERIALS AND METHODS

### Reagents and antibodies

All reagents for cell culture were purchased from Gibco BRL (Grand Island, NY, USA). LPS (*Escherichia coli* serotype 055:B5) was obtained from Sigma-Aldrich (St. Louis, MO, USA). The reagents/enzymes for reverse transcription polymerase chain reaction (RT-PCR) and oligonucleotides for electrophoretic mobility shift assay (EMSA) were purchased from Promega (Madison, WI, USA). Antibodies against phospho-/total forms of MAPKs, AMPK, and β-actin were supplied by Cell Signaling Technology (Danvers, MA, USA). Antibodies against TNF-α, HO-1, Nrf2, and lamin A were purchased from Santa Cruz Biotechnology (Santa Cruz, CA, USA) or Abcam (Cambridge, UK). The antibodies against ionized calcium binding adaptor molecule 1 (Iba1) or glial fibrillary acidic protein (GFAP) were purchased from Abcam or Invitrogen (Carlsbad, USA).

### Synthesis of MMP-8 inhibitor derivatives

Previously reported MMP-8 inhibitor derivatives (2-arylsulfonyl-1,2,3,4-tetrahydroisoquinoline-3-carboxylates and hydroxamates) [15] were synthesized through a custom synthesis service from Sehan Lab (Seongnam, Korea). We named the inhibitors as comp 1-5. Specifically, comp 1 has the same structure as the commercially available MMP-8 inhibitor (M8I) purchased from Calbiochem (La Jolla, CA, USA). We confirmed that comp 1 has the same anti-neuroinflammatory efficacy as M8I. The chemical structures of the five compounds are shown in Table 1. Synthetic procedure of comp 1-5 is included as Supplementary Materials.

### Cell cultures of microglia or astrocytes

Immortalized murine microglial cell lines, BV2 cells [32], were grown and maintained in Dulbecco’s Modified Eagle Medium containing 10% heat-inactivated fetal bovine serum, streptomycin (10 μg/ml), and penicillin (10 U/ml) at 37°C in 5% CO_2_. Primary astrocyte cultures were prepared from mixed glial cultures by modifying a previously published method [33]. In brief, after cortices were dissected from 1-day-old rats, cells were dissociated by pipetting through pores of different sizes and resuspended in minimum essential medium containing 10% fetal bovine serum, streptomycin (10 μg/mL), penicillin (10 U/mL), 2 mM glutamine, and 10 mM 4-(2-hydroxyethyl)-1-piperazineethanesulfonic acid (HEPES). Cell suspensions were plated on poly-D-lysine (1 μg/mL)-coated T75 flasks and incubated for 7 d. After the cells reached 80-90% confluency, the culture flasks were shaken at 280 rev/min for 16 h to remove microglia and oligodendrocytes. The remaining astrocytes were trypsinized and seeded onto a plastic culture plate and allowed to adhere and spread for 3 d. The cells were kept at 37°C in a humidified incubator containing 5% CO_2_. The medium was changed three times a week. Experiments were performed on astrocyte cultures between the 7th and 14th days after seeding. Astrocyte-enriched culture purity (>95%) was confirmed by staining with antibodies against the astrocyte-specific marker GFAP.

### Measurement of levels of cytokines, nitrite, and intracellular ROS

BV2 cells (1 × 10^5^ cells per well in a 48-well plate) or rat primary astrocytes (2 × 10^5^ cells per well in a 24-well plate) were pre-treated with the MMP-8 inhibitor for 1 h and further stimulated with LPS (100 ng/ml for BV2 cells, and 1 μg/ml for primary astrocytes) for 16 h. The concentrations of TNF-α and IL-6 in cell supernatants were quantified by enzyme-linked immunosorbent assay (ELISA) kits (PharMingen, San Diego, CA, USA) following the manufacturer’s instructions. Accumulated nitrite in the cell supernatant and intracellular accumulation of ROS were measured using the Griess reagent (Promega) or dichlorodihydrofluorescein diacetate (H_2_DCF-DA; Sigma-Aldrich) as previously described [13, 34].

### Animals

Adult male ICR mice (*Mus musculus*, 28-32 g, 7 weeks old) were purchased from Orient Bio (Seoul, Korea), a branch of Charles River Laboratories. All animal experiments were approved by the Institutional Animal Care and Use Committee at the School of Medicine of Ewha Womans University and at the Lee Gil Ya Cancer and Diabetes Institute of Gachon University. All animal experiments were carried out in accordance with the guidelines of the National Institute of Heath’s Guide for the Care and Use of Laboratory Animals.

### LPS-induced inflammation and administration of MMP-8 inhibitors

LPS (5 mg/kg) was administered intraperitoneally to induce neuroinflammation in male ICR mice, as previously described [13]. Comp 1 or 3 (1 or 5 mg/kg, i.p.) dissolved in vehicle solution (normal saline containing 1% DMSO), was administered daily for 4 days before LPS treatment. The brains were obtained 3 h after LPS administration and analyzed for expression of various inflammatory markers.

### Immunohistochemistry

Three hours after LPS treatment, the animals were anesthetized with avertin (500 mg/kg) and then perfused transcardially with PBS (pH 7.4) followed by ice-cold 4% paraformaldehyde. Brains were removed, incubated in fixative and a 30% sucrose solution, embedded in Tissue-Tek Optimal Cutting Temperature (OCT) compound, frozen on powdered dry ice, and cut into 20-μm sections using a cryostat (J4800AMNZ, Thermo Fisher Scientific, Waltham, MA, USA). Brain sections were treated with 1% hydrogen peroxide in PBS for 15 min and incubated for 1 h with 5% normal donkey serum in PBS containing 0.5% Triton X-100 to block nonspecific protein binding. Sections were labeled with primary antibodies against Iba1 (1:500) or GFAP (1:500), followed by labeling with the appropriate secondary biotinylated antibodies (1:200) and avidin/biotin complexes (ABC, 1:100, Vector Labs, Burlingame, CA, USA). Signals were visualized with 3,3′-diaminobenzidine (DAB) staining (0.02% DAB and 0.01% H_2_O_2_ for 5 min), rehydrated in an alcohol series, mounted using mounting medium, and photographed using a microscope (BX53T, Olympus, Japan) equipped with a DP72 camera. For quantification, the number of Iba1-positive cells (in the cortex) and GFAP-positive cells (in the cortex and corpus callosum) were counted in three different areas in the cortex and corpus callosum (in 20× images) from each mouse brain, and the average number was calculated. The total number of immunopositive cells was then expressed as the number of cells/mm^2^, as we reported previously [35].

### Reverse-transcription polymerase chain reaction (RT-PCR)

Total RNA was extracted from cultured cells or cortex using TRIzol reagent (Ambion, Foster City, CA, USA) according to the manufacturer’s instructions. For reverse-transcriptase polymerase chain reaction (RT-PCR), total RNA (1 μg) was reverse transcribed in a reaction mixture containing 1 U of RNase inhibitor, 500 ng of random primers, 3 mM MgCl2, 0.5 mM dNTPs, and 10 U of reverse transcriptase (Promega). The synthesized cDNA was used as a template for PCR using GoTaq polymerase (Promega) and primers (Table 2). Analysis of the resulting PCR products on 1% agarose gels showed single-band amplification products, which were calculated as fold change relative to control after normalization to a reference gene, GAPDH.

**Table 2:** Primers used in PCR reactions

Species	Gene	Forward primer (5’→3’)	Reverse primer (5’→3’)	Size
	**TNF-α**	CCTATGTCTCAGCCTCTTCT	CCTGGTATGAGATAGCAAAT	354 bp
	**iNOS**	CAAGAGTTTGACCAGAGGACC	TGGAACCACTCGTACTTGGGA	450 bp
Mouse	**IL-1β**	GGCAACTGTTCCTGAACTCAACTG	CCATTGAGGTGGAGAGCTTTCAGC	447 bp
	**IL-6**	CCACTTCACAAGTCGGAGGCTT	CCAGCTTATCTGTTAGGAGA	395 bp
	**GAPDH**	ATGTACGTAGCCATCCAGGC	AGGAAGGAAGGCTGGAAGAG	420 bp
	**TNF-α**	AAGTTCCCAAATGGGCTCCCT	TGAAGTGGCAAATCGGCTGAC	306 bp
	**iNOS**	GCAGAATGTGACCATCATGG	ACAACCTTGGTGTTGAAGGC	426 bp
Rat	**IL-1β**	AAATGCCTCGTGCTGTCTGACC	TCCCGACCATTGCTGTTTCCT	377 bp
	**IL-6**	TCATTCTGTCTCGAGCCCAC	GAAGTAGGGAAGGCAGTGGC	345 bp
	**GAPDH**	GTGCTGAGTATGTCGTGGAGTCT	ACAGTCTTCTGAGTGGCAGTGA	292 bp

### Transient transfection and luciferase assay

BV2 cells (1 × 10^5^ cells per well) were transfected with 1 μg of reporter plasmid DNA (ARE-luc) using the Convoy Platinum transfection reagent (ACTGene, Inc., Piscataway, NJ, USA). After 36 h of transfection, cells were treated with comp 1 or 3 before LPS (100 ng/ml) stimulation and incubated for 6 h prior to harvesting cells. Then, the luciferase assay was performed to determine the effects of comp 1 or 3 on ARE-mediated transcriptional activity.

### Electrophoretic mobility shift assay (EMSA)

BV2 cells were pretreated with comp 1 or 3 for 1 h and stimulated with LPS for 3 h. The nuclear extracts from cells were prepared as previously described [36]. Double-stranded DNA oligonucleotides containing the NF-κB, AP-1, or ARE consensus sequences (Promega or Santa Cruz Biotechnology Inc.) were end-labeled using T4 polynucleotide kinase (New England Biolabs, Ipswich, MA, USA) in the presence of [γ-^32^P]ATP. Nuclear proteins (5 μg) were incubated with a ^32^P-labeled probe on ice for 30 min, and DNA–protein complexes were resolved by electrophoresis on a 5% acrylamide gel and visualized using autoradiography. The DNA sequences of the probes were as follows: NF-κB (5′-AGTTGAGGGGACTT TCCCAGGC-3′), AP-1 (5′-CGCTTGATGAGTCAGCCGGAA-3′), ARE (5′-TGGGGAACCT GTGCTGAGTCACTGGAG-3′).

### Western blot analysis

BV2 cells were pretreated with comp 1 or 3 for 1 h prior to stimulation with LPS, and total cell lysates or nuclear proteins were prepared as described previously [36, 37]. The proteins (10-100 μg) were separated by sodium dodecyl sulfate–polyacrylamide gel electrophoresis (SDS–PAGE) and transferred to Hybond ECL nitrocellulose membranes (GE Healthcare Life Sciences, Buckinghamshire, UK). The membranes were blocked with 5% bovine serum albumin in 10 mM Tris-HCl containing 150 mM NaCl and 0.5% Tween-20 (TBST), then incubated with primary antibodies against TNF-α, HO-1, Nrf2, or lamin A (1:1000); the phospho- or total form of MAP kinases or AMPK (1:1000); or β-actin (1:5000). After thorough washing with TBST, horseradish peroxidase-conjugated secondary antibodies (1:2000 dilution in TBST; Biorad, Hercules, CA, USA) were applied, and the blots were developed using an enhanced chemiluminescence detection kit (Thermo Fisher Scientific). For quantification, the densities of specific target bands were normalized by comparison with blots for actin using ImageJ software (v 1.37; National Institutes of Health, Bethesda, MD, USA).

### Statistical analysis

Data were expressed as the mean ± S.E.M., and statistical analyses were performed using a one-way ANOVA followed by Newman-Keuls post-hoc tests or *t*-tests. Calculations were performed using GraphPad Prism software (version 4.0; GraphPad Software, Inc., La Jolla, CA, USA). A p-value less than 0.05 was considered to indicate statistical significance.

## SUPPLEMENTARY MATERIALS



## Abbreviations

AMPK: AMP-activated protein kinase
AP-1: activating protein-1
ARE: antioxidant response element
EMSA: electrophoretic mobility shift assay
ERK: extracellular signal-regulated kinase
HO-1: heme oxygenase-1
IL: interleukin
iNOS: inducible nitric oxide synthase
JNK: c-Jun N-terminal kinase
LPS: lipopolysaccharide
MAPK: mitogen-activated protein kinase
MMP: matrix metalloproteinase
NF-κB: nuclear factor-κB
Nrf: nuclear factor-E2-related factor
ROS: reactive oxygen species
TNF-α: tumor necrosis factor-α
