# Supplementary material for: Regulation of neuroinflammation by matrix metalloproteinase-8 inhibitor derivatives in activated microglia and astrocytes
Source: Oncotarget. 2017 Aug 10;8(45):78677–90. doi: 10.18632/oncotarget.20207 (PMC5667990; doi:10.18632/oncotarget.20207)
Supplement: Supplementary file 1 [file oncotarget-08-78677-s001.pdf]

Regulation of neuroinflammation by matrix metalloproteinase-8 inhibitor derivatives in activated microglia and astrocytes

SUPPLEMENTARY MATERIALS

[GENERAL PROCEDURE FOR SYNTHESIS OF MMP-8 INHIBITOR DERIVATIVES]

(R)-2-(Arylsulfonyl)-1,2,3,4-tetrahydroisoquinoline-3-carboxylic acid

(R)-1,2,3,4-tetrahydroisoquinoline-3-carboxylic acid (89 mg, 0.5 mmol) was added to a mixture of potassium carbonate (0.28 g, 2 mmol) in 25 ml of acetonitrile and 15 ml of distilled water at room temperature. After 5 min, arylsulfonyl chloride (0.6 mmol) was added. The reaction mixture was stirred for 24 hours at room temperature. This mixture was adjusted to pH 3.5 ~ 4 using 1 M HCl solution, and concentrated under reduced pressure. Then, this solution was extracted with ethyl acetate. Combined organic phase was dried with sodium sulfate. After the solid was filtered out, the organic phase was evaporated under reduced pressure, then the product was obtained.

Aryl = 4-phenoxybenzene-

Yield: 156 mg (76 %)

TLC: Rf 0.2 (ethyl acetate: hexane: methanol = 7.5:7.5:1, inactive to ferric chloride spray)

^{1}H-NMR (200 MHz) $CDCl_3$

Aryl = 4-Methoxybenzene-

Yield: 122 mg (70 %)

TLC: Rf 0.15 (ethyl acetate: hexane: methanol = 7.5:7.5:1, inactive to ferric chloride spray)

^{1}H-NMR (200 MHz) $CDCl_3$

Aryl = biphenyl-

Yield: 146 mg (74 %)

TLC: Rf 0.2 (ethyl acetate: hexane: methanol = 7.5:7.5:1, inactive to ferric chloride spray)

^{1}H-NMR (200 MHz) $CDCl_3$

Aryl = 4-chlorobiphenyl-

Yield: 152 mg (71 %)

TLC: Rf 0.25 (ethyl acetate: hexane: methanol = 7.5:7.5:1, inactive to ferric chloride spray)

^{1}H-NMR (200 MHz) $CDCl_3$

(R)-2-(Arylsulfonyl)-1,2,3,4-tetrahydroisoquinoline-3-(N-hydroxy) carboxamide

Triethylamine (0.17 ml, 1.2 mmol) and diethylchlorophospate (40 µl, 0.3 mmol) were added to a solution of (R)-2-(arylsulfonyl)-1,2,3,4-tetrahydroisoquinoline-3-carboxylic acid (0.2 mmol) in 2 ml of dichloromethane and stirred for 2 hours at room temperature, then o-benzylhydroxyl amine hydrochloride (48 mg, 0.3 mmol) was added. After 5 hours, this solution was acidified with aq. HCl and washed with aq. HCl, water and brine. The resultant organic phase was dried with anhydrous sodium sulfate. After the solid was filtered out, the filtrates were evaporated. The residues were dissolved in 5 ml of methanol, and 10 % Pd/C (cat. amount) was added. After this mixture was stirred for 5 hours at room temperature under H2 atmosphere, the solid was filtered out. The filtrates were evaporated, then the residues were purified with column chromatography (dichloromethane: methanol=20:1). The combined fractions were evaporated under reduced pressure then the product was obtained.

Aryl = 4-phenoxybenzene-

Yield: 42 mg (49 %)

TLC: Rf 0.3 (dichloromethane: methanol=15:1, active to ferric chloride spray)

^{1}H-NMR (200 MHz) DMSOd6

Aryl = 4-methoxybenzene-

Yield: 33 mg (45 %)

TLC: Rf 0.25 (dichloromethane: methanol=15:1, active to ferric chloride spray)

^{1}H-NMR (200 MHz) DMSOd6

Aryl = biphenyl-

Yield: 38 mg (46 %)

TLC: Rf 0.3 (dichloromethane: methanol=15:1, active to ferric chloride spray)

^{1}H-NMR (200 MHz) DMSOd6

Aryl = 4-chlorobiphenyl-

Yield: 37 mg (42 %)

TLC: Rf 0.35 (dichloromethane: methanol=15:1, active to ferric chloride spray)

^{1}H-NMR (200 MHz) DMSOd6

*** Ferric Chloride Spray**

Preparation: 1% ferric (III) chloride in methanol/water (1:1)
